# *Agrobacterium tumefaciens*-mediated transformation of a hevein-like gene into asparagus leads to stem wilt resistance

**DOI:** 10.1371/journal.pone.0223331

**Published:** 2019-10-07

**Authors:** Helong Chen, Anping Guo, Zhiwei Lu, Shibei Tan, Jian Wang, Jianming Gao, Shiqing Zhang, Xing Huang, Jinlong Zheng, Jingen Xi, Kexian Yi

**Affiliations:** 1 Institute of Tropical Agriculture and Forestry, Hainan University, Haikou, China; 2 Institute of Tropical Bioscience and Biotechnology/Hainan Academy of Tropical Agricultural Resource, Chinese Academy of Tropical Agricultural Sciences /Key Laboratory of Biology and Genetic Resources of Tropical Crops, Ministry of Agriculture, Haikou, China; 3 Environment and Plant Protection Institute, Chinese Academy of Tropical Agricultural Sciences, Haikou, Hainan, China; 4 South Subtropical Crops Institute, Chinese Academy of Tropical Agricultural Sciences/ Zhanjiang City Key Laboratory for Tropical Crops Genetic Improvement, Zhanjiang, Guangdong, China; National University of Kaohsiung, TAIWAN

## Abstract

Asparagus stem wilt, is a significant and devastating disease, typically leading to extensive economic losses in the asparagus industry. To obtain transgenic plants resistant to stem wilt, the hevein-like gene, providing broad spectrum bacterial resistance was inserted into the asparagus genome through *Agrobacterium tumefaciens*-mediated transformation. The optimal genetic transformation system for asparagus was as follows: pre-culture of embryos for 2 days, inoculation using a bacterial titre of OD600 = 0.6, infection time 10 min and co-culturing for 4 days using an Acetosyringone concentration of 200 μmol/L. Highest transformation frequencies reached 21% and ten transgenic asparagus seedlings carrying the hevein-like gene were identified by polymerase chain reaction. Moreover, integration of the hevein-like gene in the T1 generation of transgenic plants was confirmed by southern blot hybridization. Analysis showed that resistance to stem wilt was enhanced significantly in the transgenic plants, in comparison to non- transgenic plants. The results provide additional data for genetic improvement and are of importance for the development of new disease-resistant asparagus varieties.

## Introduction

Asparagus (*Asparagus officinalis L*.), known as the “King of Vegetables”, is among the most popular nutrient-rich and healthy vegetables because of its unique texture, taste and high nutrient content [1]. Asparagus has various bioactive properties [2]. However, research on development of improved Asparagus germplasm in China is lacking. Stem wilt (*Phoma asponaqi Sacc*) of asparagus is a severe disease that leads to huge economic losses. Currently, transgenic breeding of Asparagus has not been exploited and only three reports regarding transgenic research on asparagus have been published. Delbreil [3] and Cabrera [4] have successfully implemented transgenic technology via the “gene gun” by cultivating regenerated asparagus plants with *A*. *tumefaciens*, Mukhopadhyay [5] used electroporation to observe the transgenic characteristics in the microbial community and callus derived from protoplasts, and Garrison [6] considered transferring the required genes into asparagus to enhance resistance to herbicide and insect infestations.

The hevein gene, also called the “Rubber tree agglutination factor gene¨, encodes a small protein that is rich in Gly and Cys and mainly acts in rubber particle agglutination in latex [7–8]. In addition, hevein and hevein-like proteins belong to a group of antimicrobial peptides, whose action mechanism is associated with the cell wall chitin of pathogens, and they can quickly penetrate pathogenic hyphae and destroy fungal cell membranes, thus causing leakage of the cytoplasm, eventually leading to rupture of the mycelial tip. As a result, they provide broad-spectrum resistance to bacteria, fungi and other pathogens [9–12].

Therefore, we developed a genetic transformation system for asparagus and applied transgenic technology to obtain transgenic plantlets through *A*. *tumefaciens*-mediated transformation. Analysis of disease resistance and determination of physiological indexes confirmed that the transgenic lines represented a new germplasm source for breeding high yielding, disease-resistant Asparagus varieties.

## Materials and methods

The diploid asparagus cultivar “Jing Kang 701” was used to develop the transformation protocol.

pBI121 and pCAMBIA3300 expression vectors and the pUC57-hevein-like vector were available within our laboratory and the bacterial strains used were *A*. *tumefaciens* EHA105 and *E*. *coli* DH5α. Ligase, restriction enzymes and Taq polymerase were purchased from New England Biolabs (Beijing), ThermoFisher, and Takara, respectively. Kits were purchased from OMEGA. Synthesis and sequencing were handled by Sangon Biotech. The hevein-like target gene sequence is registered under GenBank Accession No: U40076.1. See the following website for the detail protocols: http://dx.doi.org/10.17504/protocols.io.68bhhsn.

### Vector construction

**Fig 1:**
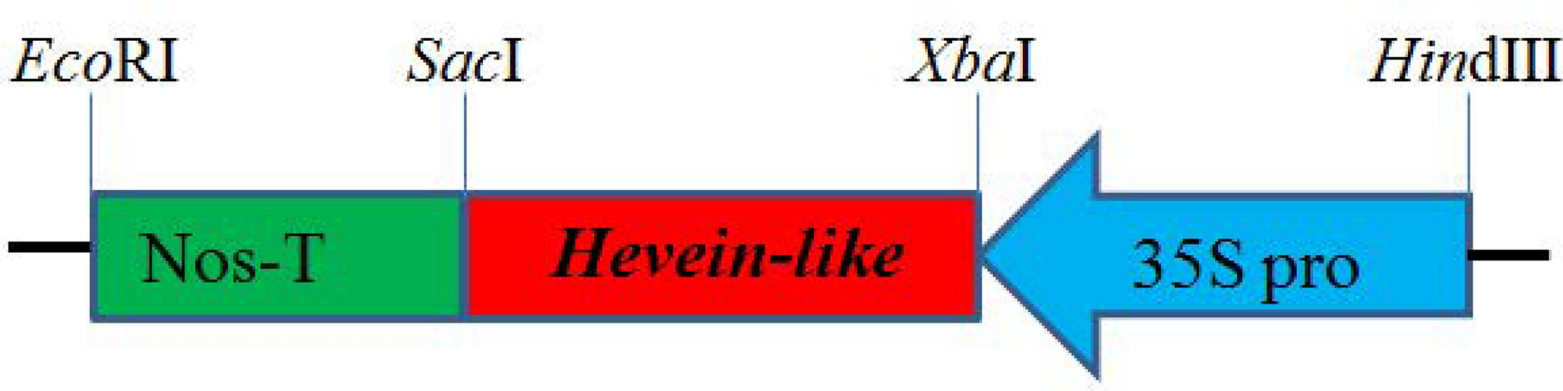
The fragments cloned of pBI121-hevein-like vector.

The fragments cloned of the pBI121-hevein-like vector was shown in Fig 1. Extraction and purification of pBI121 and the hevein-like carrying plasmids were performed using kits according to the manufacturer’s instructions. We used *Xba*I and *Sac*I for enzyme digestion and performed gel extraction using a kit (OMEGA). The P1 primer was used to add *Xba*I and *Sac*I cleavage sites to the ends of the hevein-like target gene.

F: 5’ TGCTCTAGAATGAAATACTGTACTATGTTTAT 3’

R: 5’ CGAGCTCTCAGTTGGCACCGC 3’

**Fig 2:**
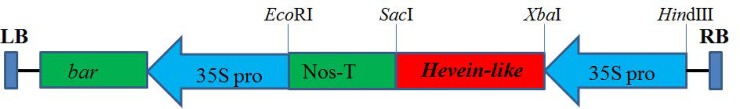
The fragments cloned of the plant expression vector-pCAMBIA3300-35S-hevein-like-NOS.

The pCAMBIA3300-35S-hevein-like-NOS vector (Fig 2) was constructed using the purified hevein-like gene fragment. The P2 primer below was used for PCR verification, and the construction was completed upon verification.

F: CCCAAGCTTCATGGAGTCAAAGATTCAAATAG

R: GGAATTCCCCGATCTAGTAACATAGATGAC

### Glufosinate resistance

A total of 20, 0.6 cm^2^ blocks of asparagus embryos were placed in transformation media containing different concentrations of glufosinate (PPT) (0, 5, 10, 20, 40 and 80 mg/l PPT), and the survival rate of asparagus embryos was recorded after 20 days.

### Preculture

Asparagus embryos measuring 0.6 cm^2^ each were placed in preculture medium for 2 days at 26°C. The formula of the culture medium was: Murashige and Skoog (MS) medium, 4% sucrose, 800 mg/l Gln, 500 mg/l acid-hydrolyzed casein, 0.70 mg/l ancymidol, 0.10 mg/l naphthaleneacetic acid (NAA), and 0.50mg/l kinetin, pH 5.8.

### Preparation of *A*. *tumefaciens* cultures

A total of 20 μL of an *Agrobacterium tumefaciens* strain containing the pCAMBIA3300-35S-hevein-like-NOS plasmid was grown on yeast extract peptone (YEP) solid medium (containing 50 m/l Kanamycin and 100 mg/l Rifampin). The medium was placed in stationary position and turned upside down for 30 min to initiate cultivation at a temperature of 28°C for 2 days. A single colony was inoculated in 50 mg/l Kanamycin (Km) and 100 mg/l Rifampin (Rif) containing YEP liquid medium and cultivated under agitation at 250 rpm at 28°C. Cultures (OD600 about 0.55) were centrifuged for 8 min at 5500 rpm and re-suspended in liquid transformation medium.

### Inoculation

The OD 600 nm of the bacterial cultures was adjusted to 0.2, 0.4, 0.6 and 0.8. The cultivated embryos were placed in a sterile 150 mL Erlenmeyer flask containing 50 mL liquid media supplemented with AS of different concentrations (0, 50, 100 and 200 μmol/l). Embryos were incubated for 5, 10, 20 and 40 min at 26°C and shaken at a frequency of 150 rpm.

### Co-cultivation

The inoculated embryos were wiped dry and transferred to solid medium for culturing in darkness at 26°C for 2, 4, 6 and 8 days.

### Removal of bacteria and selection of transformed embryos

The co-cultured embryos were treated with 200 mg/l Timentin for 10 min at 100 rpm to remove the bacteria. Embryos were washed with sterile distilled water, wiped dry, transferred to a solid transformation medium containing glufosinate (PPT) and Timentin for culture under light conditions at 26°C. Growth of the embryos was observed and statistically analyzed in triplicate (80 embryos were used each time).

Transformation percentage of resistant embryos = number of resistant embryos/total number of embryos *100%

### DNA extraction and confirmation of transformation

Selected embryos were placed in a seedling culture medium. The seedlings produced were transplanted to a prepared nutritional substrate after induction until testing to detect the presence of the transgene. A kit method was adopted to extract DNA from putatively transformed asparagus lines. The P2 primer mentioned in vector construction was used for PCR verification. Moreover, integration of the hevein-like gene was further confirmed by southern blot hybridization. Genomic DNA of T1 generation transgenic plant leaves was used as template, genomic DNA of non-transgenic plant leaves was used as negative control, and plasmid pCAMBIA3300-35S-hevein-like-NOS DNA was used as positive control. Genomic DNA was digested overnight at 37°C using *EcoR*I and *Hind*III, separated on an 0.8% agarose gel and transferred onto a Hybond-N membrane (Pharmacia, USA). The membrane was hybridized with the hevein-like-gene CDS DNA probe, which was random primed, labeled with Digoxigenin-11-dUTP using DIG-High Prime (Roche). Hybridization (at 42°C) and detection were performed following the instruction manual of the DIG High Prime DNA Labeling and Detection Starter Kit I (Roche, USA).

### Disease resistance and physiological index determination in transgenic plants

The stem wilt disease resistance of transgenic asparagus plants was calculated using the disease index described by Yang [13]. The infective strain was the virulent strain BT05 screened in the laboratory. BT05 was prepared as 10^6^/mL bacterial suspensions for spraying with the bacterial suspension to inoculate. Three transgenic plants were treated and three non-transgenic plants of the same seedling age were compared. Three replicates were carried out. Inoculated samples were left under high humidity for 48 hours, and then the plastic cover was removed, allowing natural infection at a temperature of 25°C to 28°C. On the 8th day after inoculation, the disease incidence of each treatment was observed and the disease level of each replicate was determined according to disease level criteria, and the DI (disease index) was calculated according to the disease level.

### Disease level criteria

Grade 0: no disease; Grade 1: the disease area accounts for less than 10% of the canopy area; Grade 2: the disease area accounts for 11%-30% of the canopy area; Grade 3: the disease area accounts for the crown area 31%-50%; Grade 4: The disease area accounts for more than 50% of the canopy area.

D I = ∑ (plant number of disease grades × disease level value) / (number of total plants per replicate × 4) × 100;

Immunity: DI = 0; High resistance: 0<DI≤10; Medium resistance 10<DI≤30; Resistance: 30<DI≤50; Susceptibility: DI>50

Presence of Malondialdehyde (MDA), Superoxide dismutase (SOD), Catalase (CAT) and Phenylalanine ammonia lyase (PAL) level were determined in inoculated leaves by the method of Han [14].

### Data analysis

All the experiments were carried out in triplicate using a completely randomized design, asparagus embryos number of PPT resistance test was 20 in each repeat, whereas embryos number of each repeat was 80 in other tests. SPSS19.0 and Excel 2007 were employed for statistical analysis [15] and significantly different levels of disease resistance and physiological index between transgenic and control lines were calculated using SPSS19.0 software.

## Results

### Construction of vector

#### pBI121-hevein-like

**Fig 3:**
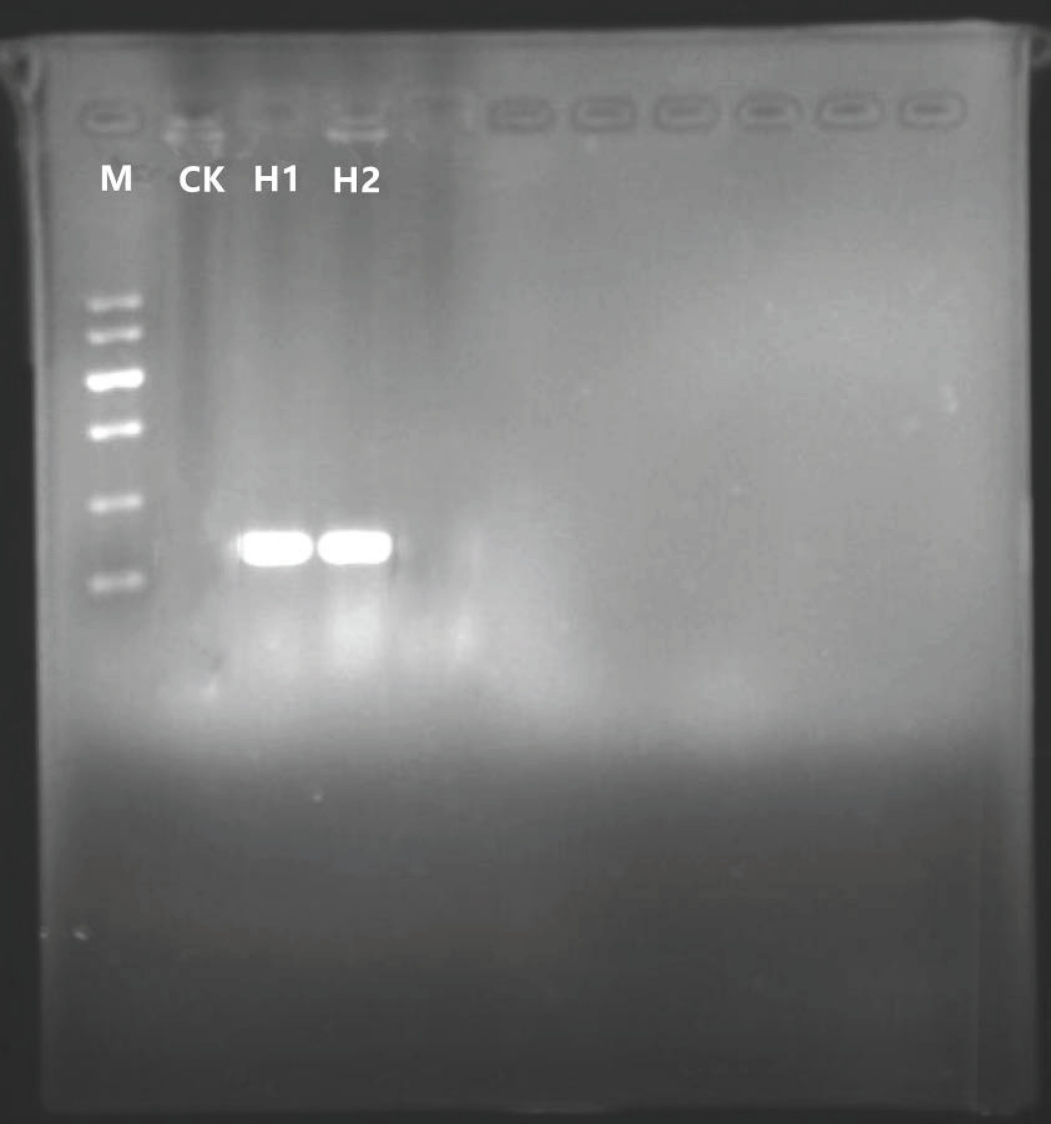
PCR detection of *E*.*coli* colonies carrying the pBI121-hevein-like vector. M: DNA Marker II; CK: H_2_O blank control; H1, H2: positive samples.

Plasmid pBI121 was digested with *Xba*I and *Sac*I, followed by agarose gel electrophoresis and purification of the pBI121 large fragment. At the same time, primers were designed to carry out PCR amplification of the hevein-like gene in plasmid pUC57-hevein-like. *Xba*I and *Sac*I restriction sites were added to both ends of the hevein-like gene amplification product, and then the PCR amplification product was subjected to DNA digestion. Finally, the large fragment of plasmid pBI121 and the hevein-like gene were ligated and the products transformed into *E*.*Coli* DH5α. Two single colonies were randomly picked for confirmation of the presence of the hevein-like gene by colony PCR. The target gene fragments were discrete with no background fragments, showing a fragment size of 276 bp, the expected size for the hevein-like gene (Fig 3).

#### pCAMBIA3300-35S-hevein-like-NOS

**Fig 4:**
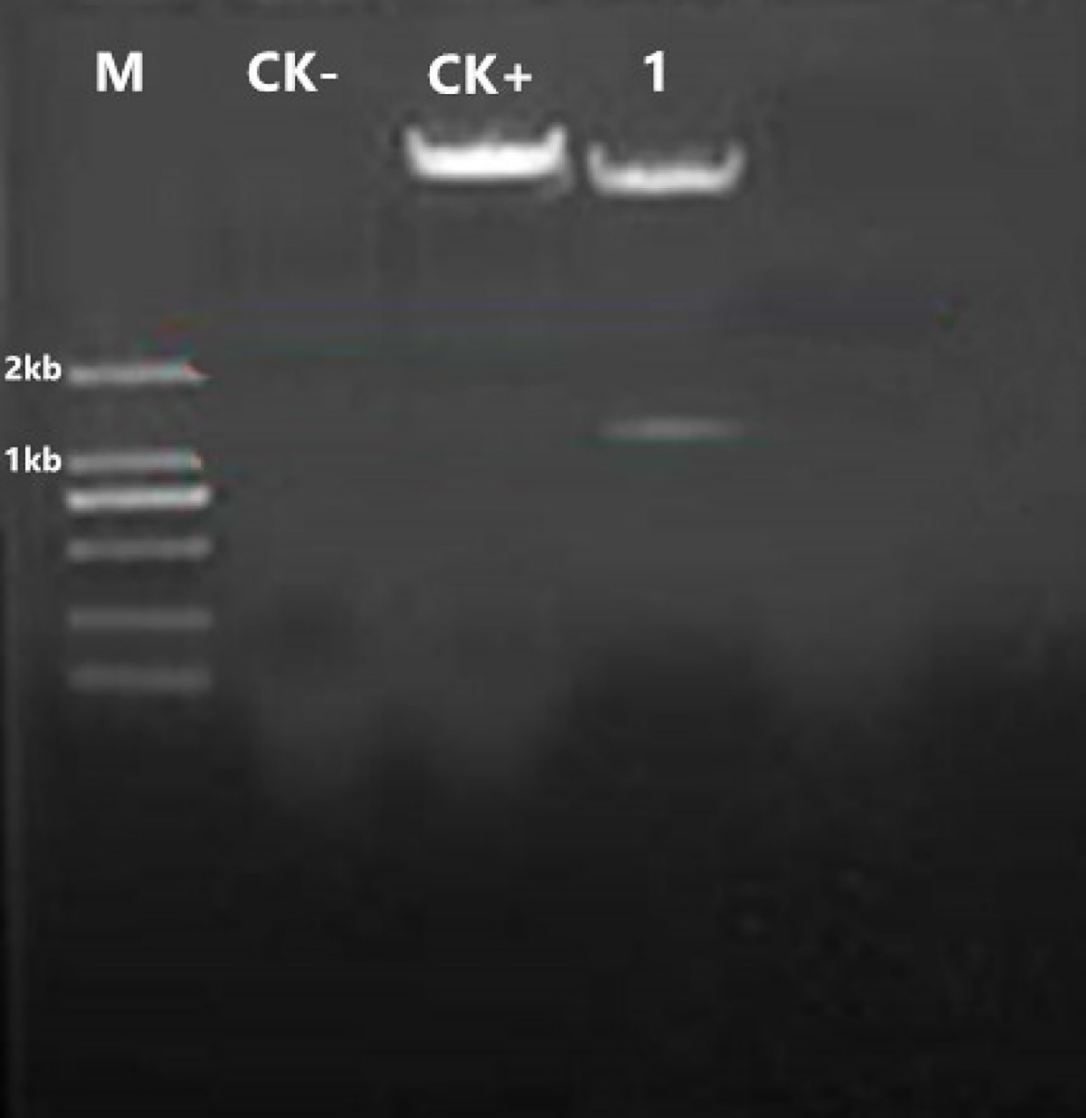
Detection of double restriction enzyme digestion for pCAMBIA3300-35S-hevein-like-NOS vector. M: DNA Marker D2000; CK-: H_2_O blank control; CK+: pCAMBIA3300-35S-hevein-like-NOS vector; 1: the size of pCAMBIA3300-35S-hevein-like-NOS vector after double restriction enzyme digestion.

**Fig 5:**
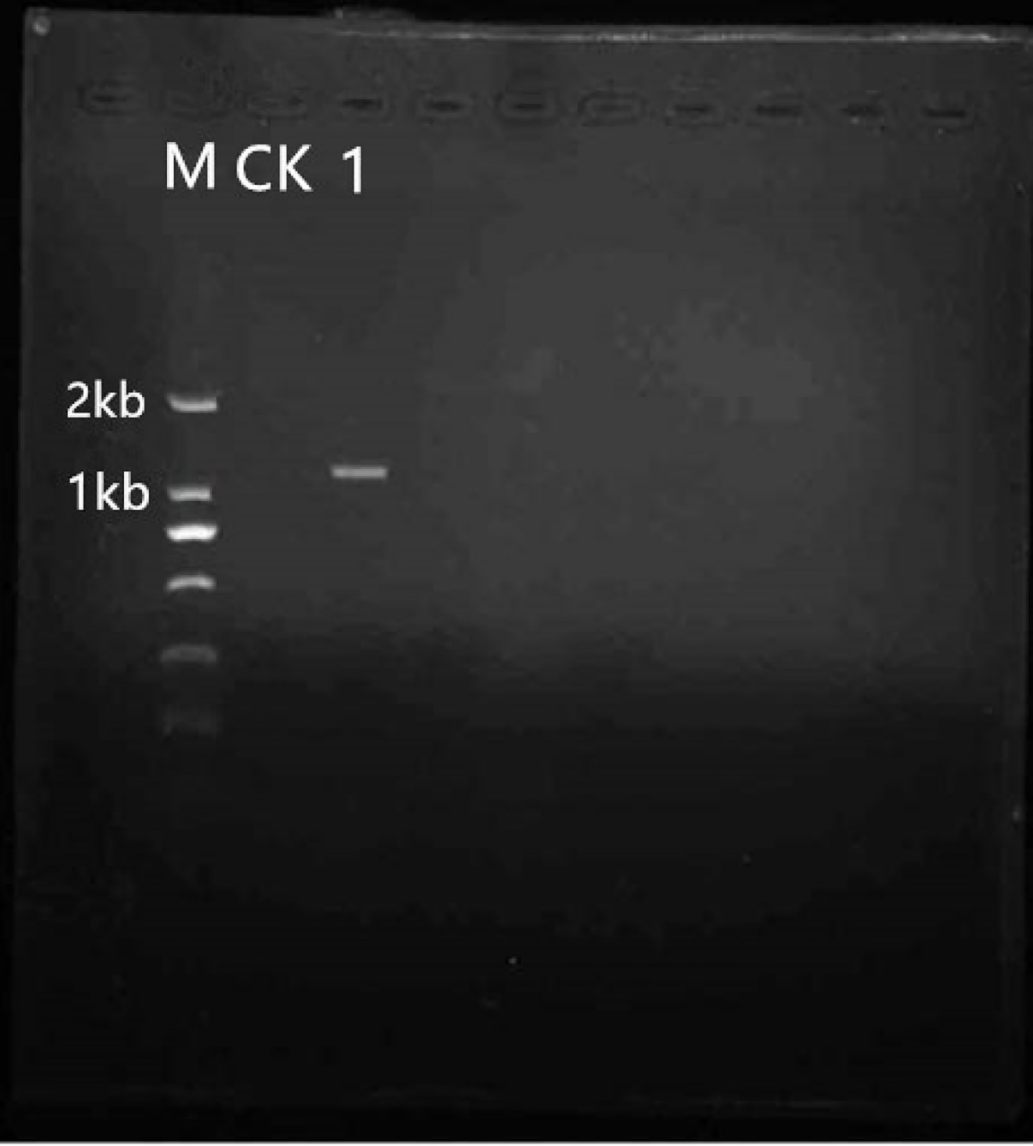
PCR detection for *A*.*tumefaciens* transformation. M: DNA Marker D2000 CK: H_2_O blank control 1: *Agrobacterium*.

Plasmid pBI121-hevein-like and plasmid pCAMBIA3300-35S-hevein-like-NOS were digested with *Hind*III and *EcoR*I, respectively, and then the fragments of plasmid pBI121-hevein-like and plasmid pCAMBIA3300-35S- hevein-like-NOS were separately purified. The fragments were ligated and the products were transformed into *E*.*coli* DH5α. Finally, the constructed plasmid pCAMBIA3300-35S-hevein-like-NOS was purified and verified by *Hind*III and *EcoR*I double digestion. The fragment size of the target band was approximately 1.1 kb consistent with the coding sequence of the target gene (Fig 4). Plasmid extraction was performed on *E*.*coli* DH5α carrying the pCAMBIA3300-35S-Hevein-like-NOS plasmid, then transformed into *A*. *tumefaciens* EHA105, and finally verified by colony PCR. The fragment size of the target band on the gel was approximately 1.1 kb and was consistent with the target gene 35S-hevein-like-NOS construct (Fig 5).

### Influence of different glufosinate concentrations on growth of asparagus embryos

**Fig 6:**
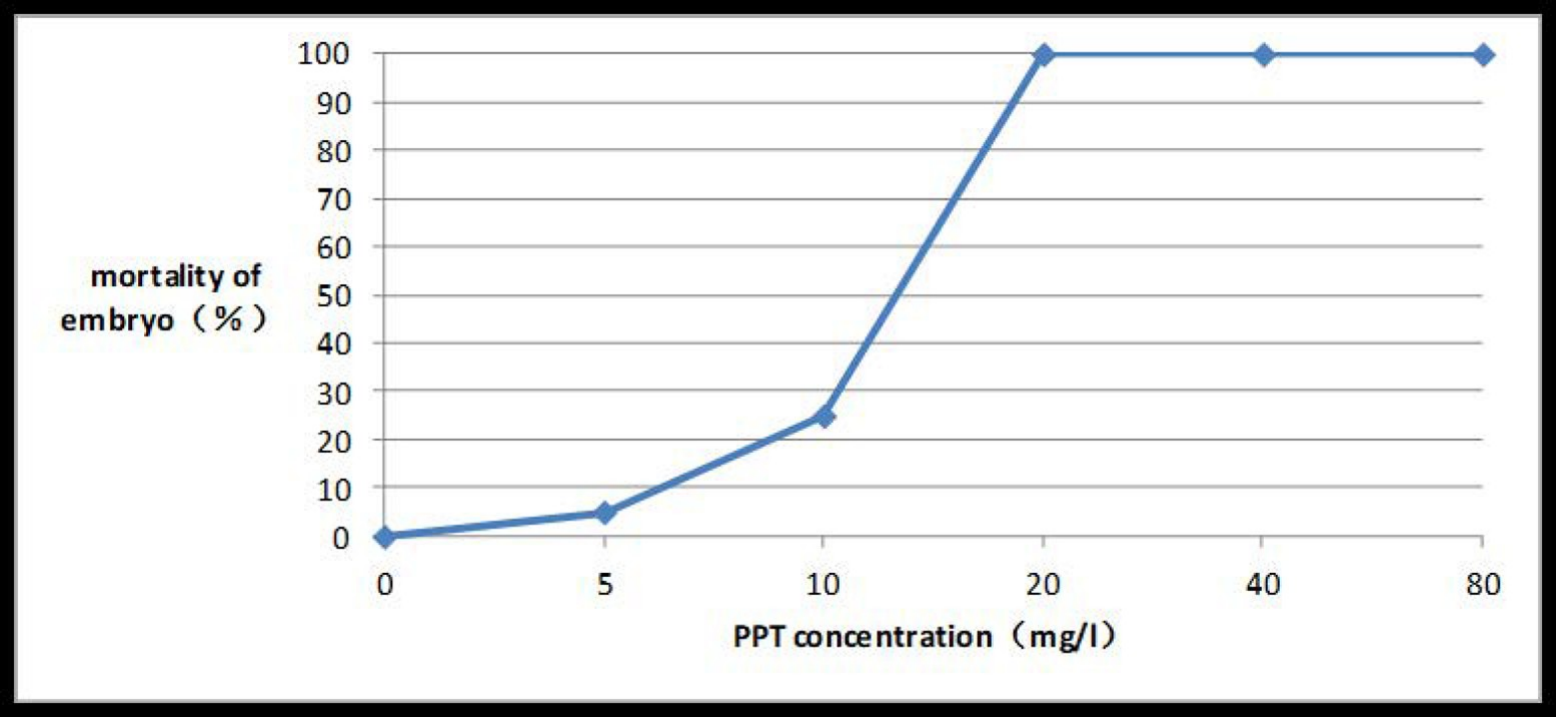
Embryo death rate under different PPT concentrations.

**Fig 7:**
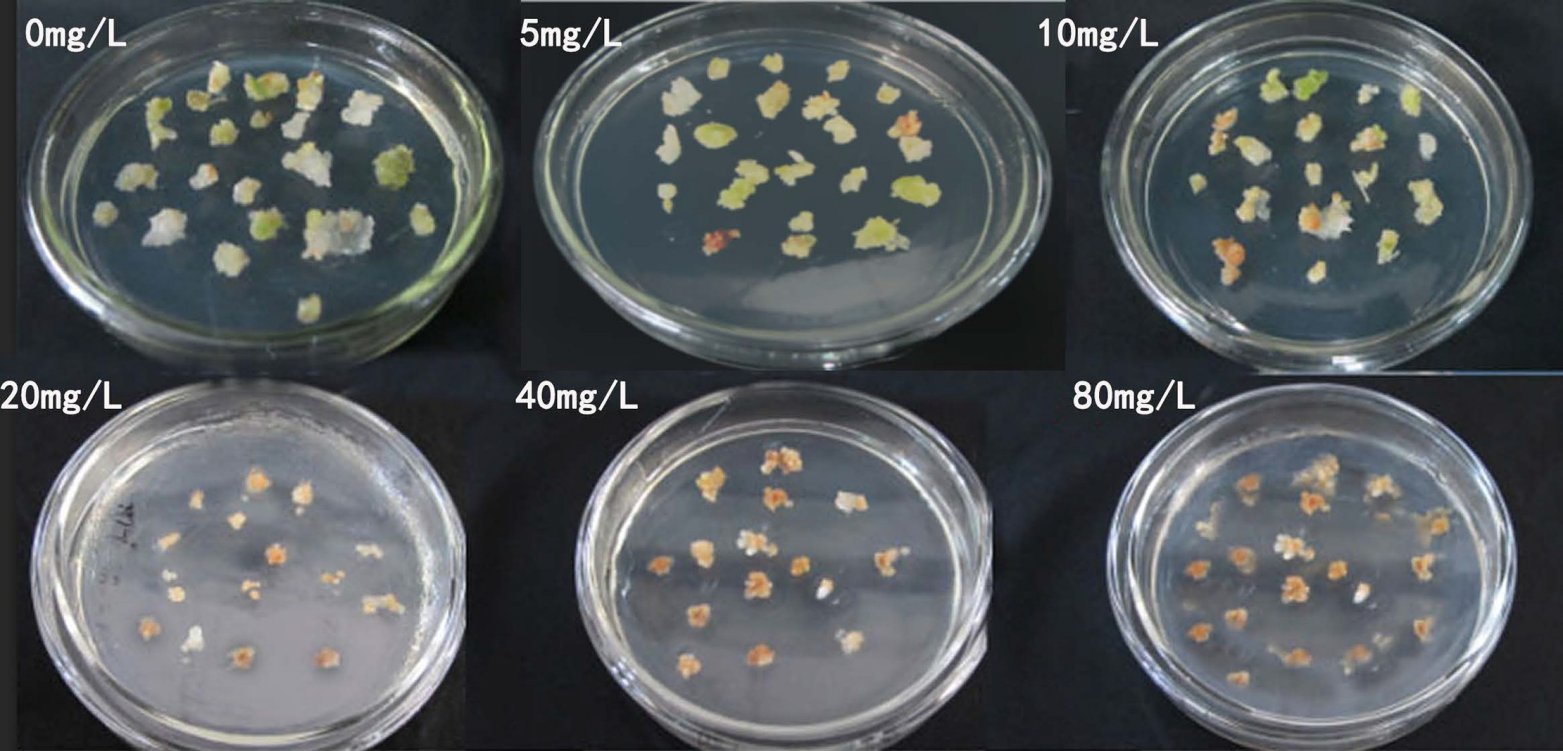
Examples of embryo death under different PPT concentrations.

Before PPT concentration reached 20 mg/l, the mortality of the embryos was positively correlated with the PPT concentration. As the concentration rose, the embryo apparently shrank, discolored, and eventually died. If the PPT concentration was equal to or greater than 20 mg/l, embryo mortality reached 100% (Figs 6 and 7).

### Influence of inoculation time and OD of *A*. *tumefasciens* cultures on embryo transformation rate

**Fig 8:**
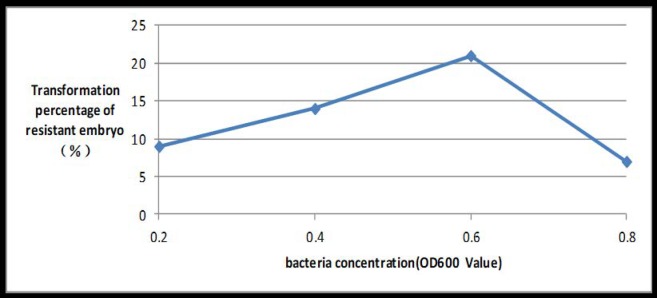
Induction rate of PPT-resistant embryos under different ODs of *Agrobacterium* suspensions.

**Fig 9:**
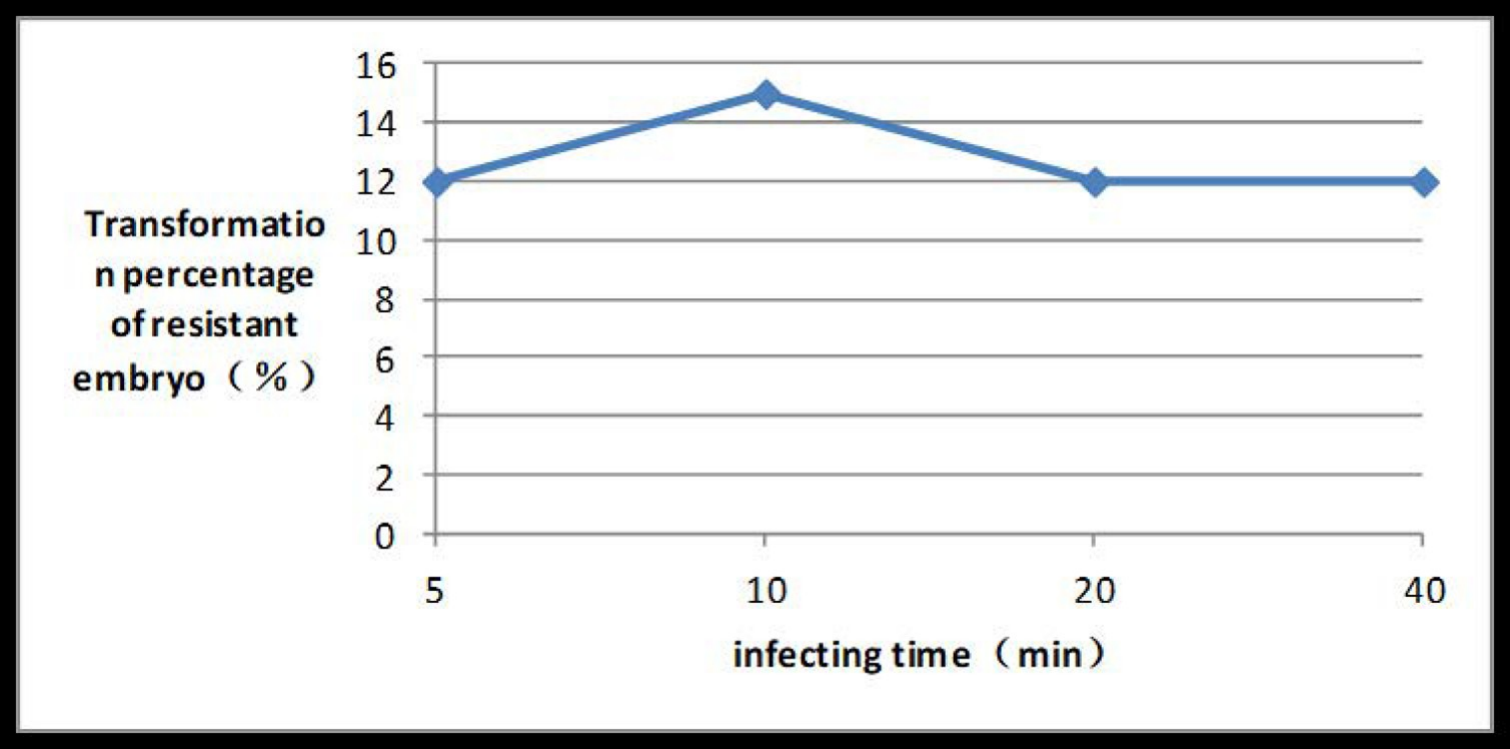
Induction rate of PPT-resistant embryos at different *Agrobacterium* infection times.

If the OD of the bacterial suspension reached 0.6 at a wave-length of 600 nm, the transformation rate peaked at 21%. If the inoculation time reached 10 min, the transformation rate peaked at 15% (Figs 8 and 9).

### Influence of different co-culture times on embryo transformation rate

**Fig 10:**
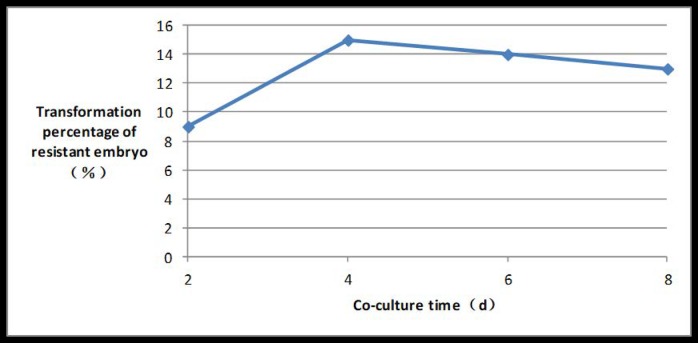
Induction rate of PPT-resistant embryos under different co-culture times.

The embryo transformation rate rose initially and then decreased as the length of time of co-culture increased. The embryo transformation rate peaked at 15% if the co-culture time reached 4 days (Fig 10).

### Influence of different concentrations of AS on embryo transformation rate

**Fig 11:**
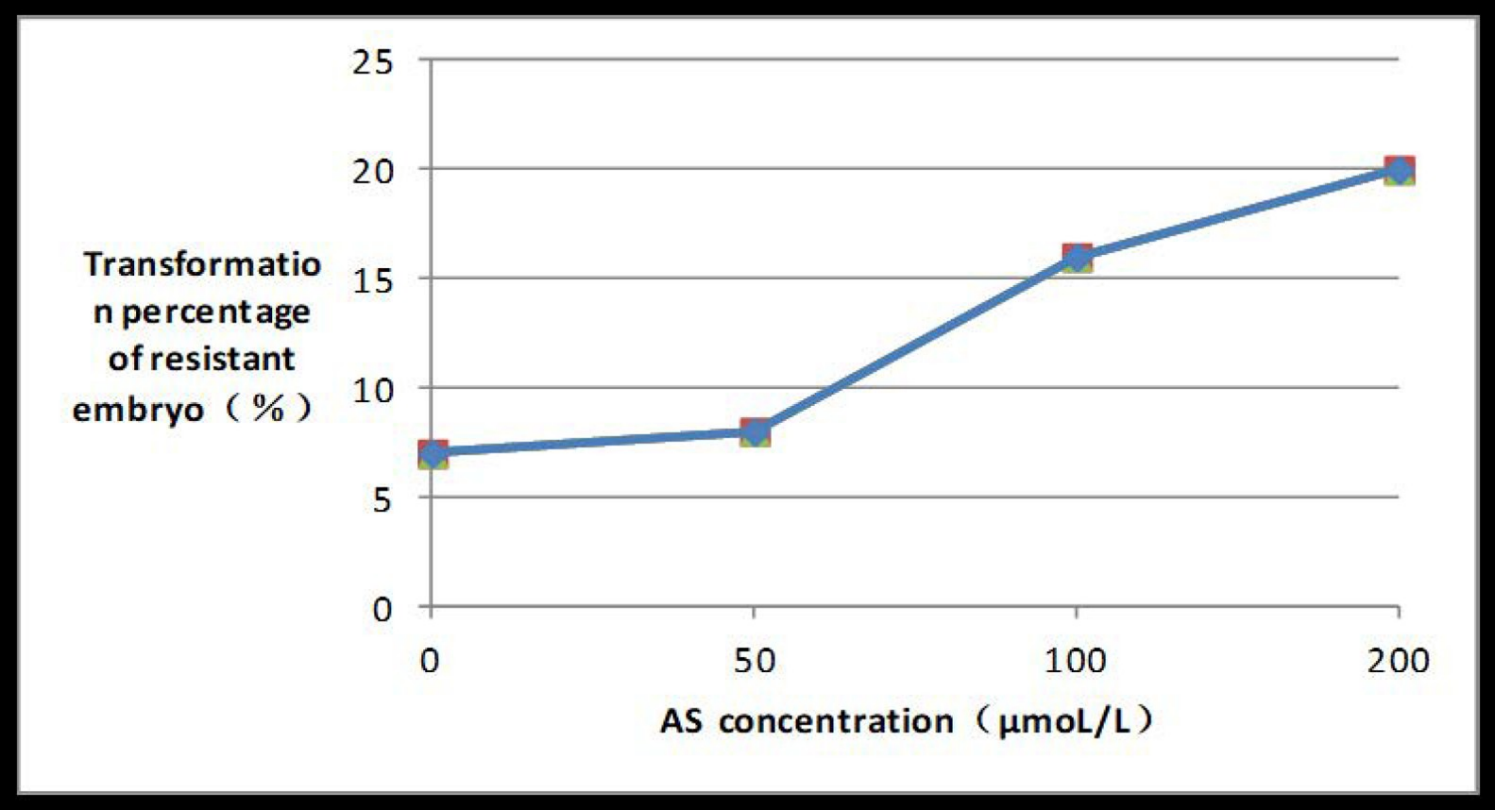
Induction rate of PPT-resistant embryos under different AS concentrations.

The embryo transformation rate was positively correlated with the AS concentration, and peaked at 20% if AS concentration reached 200 μmoL/l (Fig 11).

### Detection of transgenic asparagus seedlings

**Fig 12:**
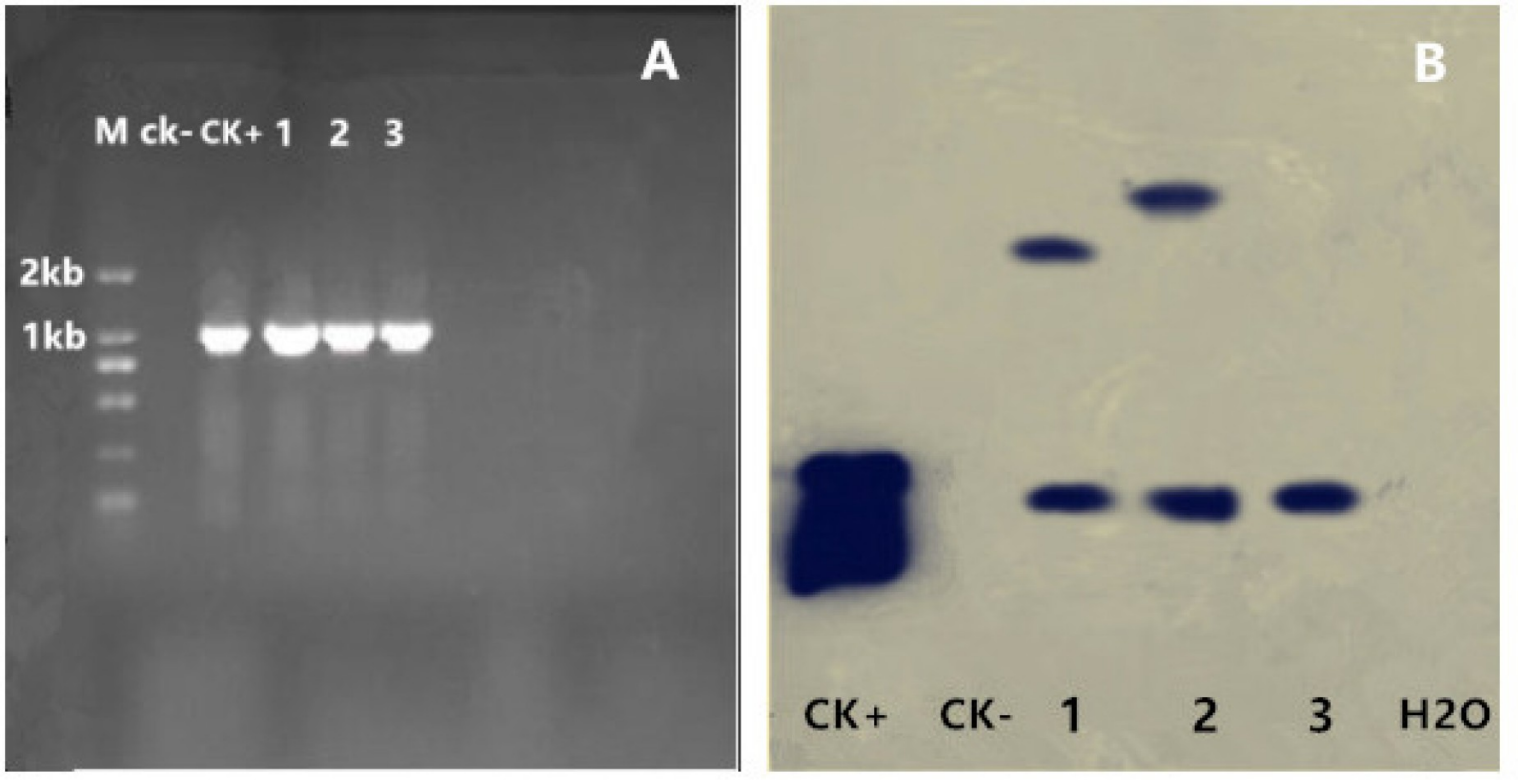
Molecular confirmation results of transgenic plants. (A) Results of PCR detection of transgenic plants M: D2000 DNA Marker CK-: Non-transgenic plants CK+: pCAMBIA3300-35S-hevein-like-NOS vector 1, 2, 3: Transgenic plants (B) Results of southern blot hybridization of T1 generation transgenic plants CK+: pCAMBIA3300-35S-hevein-like-NOS vector CK-: Non-transgenic plants 1, 2, 3: T1 generation transgenic plants.

**Fig 13:**
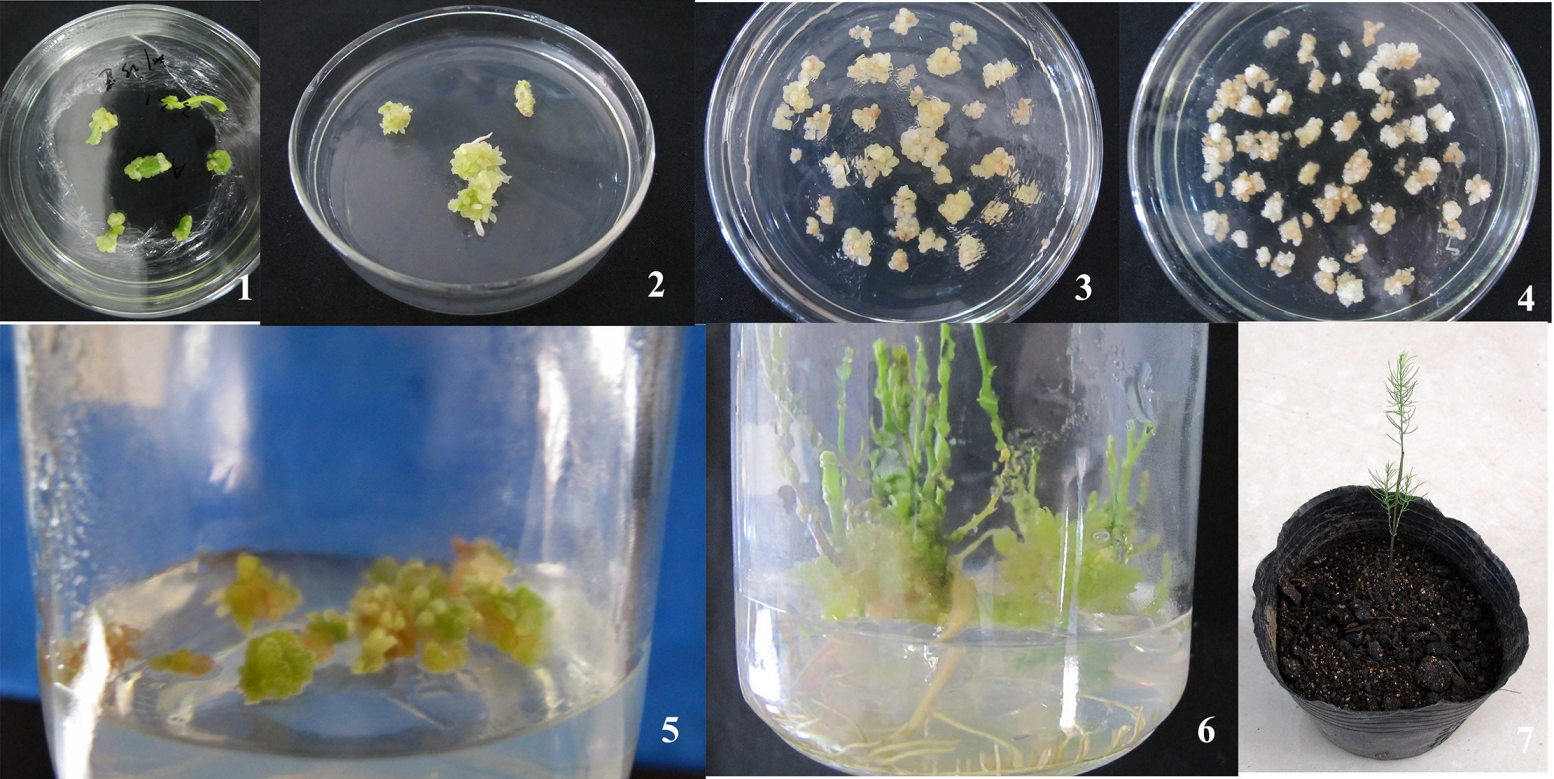
Process of producing transgenic Asparagus plants. 1: Induction of embryogenic callus; 2: Induction of embryos; 3: *A*. *tumefaciens*-mediated genetic transformation of asparagus embryos; 4: Screening of *A*. *tumefaciens*-mediated genetic transformation of embryos; 5: Induction of screened transformants; 6: Production of transgenic seedlings; 7: Outdoor transplantation.

The target fragment size of the PCR reactions used to identify transgenic asparagus seedling was approximately 1.1 kb, identical with the positive control and consistent with the size of 35S-hevein-like-NOS element (Fig 12). By this method, 10 transgenic asparagus seedlings were identified. See Fig 13 for the complete transgenesis flowchart. Moreover, three T1 generation transgenic plants were selected for southern blot hybridization. All plants showed a hybridization signal, proving that the exogenous gene Hevein-like had been integrated into the genome. The hybridization results were shown in Fig 12, where the first and second plants showed 2 bands, whereas the third plant showed only a single band.

### Disease resistance identification and physiological index determination in transgenic plants

Disease index was calculated according to the incidence and degree of severity of infection of each treatment on the 8th day after inoculation, the results are shown in Table 1. The disease index of transgenic plants was 42, but for non- transgenic plants it was 75. The disease resistance was graded according to the disease index, transgenic plants were resistant (30<DI<50), while non-transgenic plants were susceptible (50≤DI). The disease resistance level of transgenic plants was higher than that of non-transgenic plants, showing strong statistical significance.

**Table 1:** Disease index of transgenic and non-transgenic asparagus.

	Transgenic plants	Non-transgenic plants
Disease index	42± 2.35**	75±2.78

Note

“**” indicate significant difference between treatment and control at 0.01 level.

The physiological index for asparagus leaves was determined after resistance had been confirmed; the results are shown in Table 2. Compared with non-transgenic plants, MDA content negatively correlated with disease resistance, the reason is that MDA is a strong oxidant and the main product of membrane lipid peroxidation decomposition. The increase in MDA content can damage plant cells. MDA content therefore reflected the weak ability of plants to respond to adverse conditions and was decreased, whereas the activity of SOD, CAT and PAL (positively correlated with disease resistance) was increased and the observed differences were statistically highly significant, confirming that the disease resistance of the transgenic plants was enhanced.

**Table 2:** Effect of stem wilt on physiological index in transgenic and non-transgenic asparagus.

physiological index	MDA (μmol/g)	SOD(u/g)	CAT(u/g)	PAL(u/g)
Non-transgenic plants	7.05±0.02	48.42±0.05	47.65±0.02	7.54±0.08
Transgenic plants	5.16±0.03**	74.39±0.03**	87.26±0.06**	11.87±0.05**

Note

“**” indicate significant difference between treatment and control at 0.01 level.

## Discussion

The OD of *A*. *tumefaciens* suspension, inoculation time, AS concentration, embryo type, and co-culture times all influenced transformation mediated by A. tumefaciens. Different plants had different sensitivity to Agrobacterium concentration, and appropriate Agrobacterium concentration and infection time were very important to improve the transformation rate. Agrobacterium concentration OD600 for orchid [16], potato [17], kiwifruit [18] and switchgrass [19] transformation methods was 0.8, 0.5, 0.5 and 0.8 respectively, and infection time was 30 min, 8 min, 10 min and 20 min respectively. Whereas Agrobacterium concentration OD600 and infection time in this experiment was 0.6 and 10 min respectively. If the Agrobacterium concentration was too low, or the infection time was too short, then the quantity of bacteria was insufficient, and the interaction time between bacteria and embryos was too short, resulting in a decrease in transformation efficiency. If *Agrobacterium* concentration is too high and infection time too long a large number of Agrobacterium bacteria gather around the embryos for a long time. Due to nutrition, space and other reasons, Agrobacterium will produce a large amount of harmful metabolites around the embryos, so that the toxic effect of Agrobacterium on the plants is greater than the transformation effect, resulting in a large number of embryo deaths. Co-culture time played an important role in improving transformation rate of Asparagus since the T-DNA can only be transferred to plants after the contact exists for a period of time. However, when contact time was too long, Agrobacterium would overgrow, eventually leading to embryo death. Co-culture time of orchid [16], potato [17], kiwifruit [18] and switchgrass [19] was 4d, 2d,2d and 3d respectively, while co-culture time in this experiment was 4d. The addition of AS can greatly promote T-DNA transformation to plants, but different plants have different sensitivities to AS. The AS concentration of orchid [16], kiwifruit [18] and switchgrass [19] was 200μmol/L, 100μmol/L and 200μmol/L respectively, while AS concentration in this experiment was 200μmol/L. Moreover, the transformation rate apparently also varied with these factors. Successful transformation mediated by A. tumefaciens has been reported previously and for orchid and potato under the mediation reached 33.6%[16] and 37.3%[17], respectively; whereas the transformation rate of Chinese kiwifruit “Hongyang”, in which the LJAMP2 gene was successfully introduced, only reached 5.11% [18], and transformation efficiency of switchgrass was only 5.6%[19]. In this research, an optimal asparagus genetic transformation system with a transformation rate of 21% was developed. The results are consistent with previous reports [20–22], however the transformation rate in this study is slightly higher.

The major factors affecting transformation rate of A. tumefaciens were embryo type, A. tumefaciens strains and the selective agent. Embryos played an important role in the transformation efficiency of A. tumefaciens. According to the study by Hernalsteens [20], embryogenic callus was used as the infection target of Agrobacterium in this experiment, thus the effect was remarkable. Different strains of Agrobacterium have different infectivity, and transformation experiments of asparagus have mainly used A. tumefaciens strains C58[20–21] and AGL1Gin[22], while A. tumefaciens strain EHA105 was used in this experiment, and the transformation rate obtained was slightly higher, indicating that the transformation effect of EHA105 was higher than that of C58 and AGL1Gin. The regeneration capacity of plants was also dependent on the type of selective agent. According to Zhai [23] and Duan [24], if an antibiotic was used as the selective agent, the mortality of plants rose significantly. For this study, PPT was used as the selective agent. Therefore, screening new herbicide-resistant asparagus materials is possible.

MDA content was negatively correlated with plant disease resistance, while SOD, CAT and PAL contents were positively correlated with plant disease resistance [14]. It can be seen from the experiment that transgenic asparagus acquired a higher ability to protect the cell membrane after pathogen infection, and the MDA content was reduced, showing resistance to fungal infection. The concentration of SOD and CAT in transgenic asparagus increased significantly, indicating that the transgenic asparagus had a higher ability to remove reactive oxygen species, thereby weakening the toxic effect on cell membrane and other active intracellular substances, thus improving the resistance of asparagus to fungal infection. By affecting the accumulation of polyphenols, alkaloids and other important secondary metabolites, PAL can affect the stress resistance of plants. The PAL content in transgenic asparagus increased significantly, indicating that it had higher fungal resistance. The research results were consistent with those of Song, where SOD, CAT and PAL contents of resistant rapeseed cultivars were higher and their MDA content was lower [25].

## Conclusion

Ten transgenic asparagus seedlings with the hevein-like and bar genes were obtained; their disease resistance was enhanced and was highly significant in relation to non-transgenic plants thereby laying a foundation for the cultivation of new disease-resistant asparagus varieties.

## Supporting information

S1 File Data set. (XLSX)
